# Emergent symbiont strains provide thermally robust protection against co-evolved and novel parasitoids of introduced pea aphids

**DOI:** 10.1093/ismejo/wrag098

**Published:** 2026-04-17

**Authors:** Vilas Patel, Roy A Kucuk, Benjamin R Haines-Eitzen, Jacob A Russell, Kerry M Oliver

**Affiliations:** Department of Entomology, University of Georgia, Athens, GA 30602, United States; Department of Entomology, University of Georgia, Athens, GA 30602, United States; Department of Entomology, University of Georgia, Athens, GA 30602, United States; Department of Biology, Drexel University, Philadelphia, PA, 19104, United States; Department of Entomology, University of Georgia, Athens, GA 30602, United States

**Keywords:** climate change, facultative symbiont, bacteriophage, heat wave, endosymbiont, symbiosis, biological invasion, parasitoid, thermal tolerance, *Hamiltonella defensa*

## Abstract

Climate change and biological invasions pose synergistic threats; however, organisms may rapidly adapt through microbial symbiosis. We investigated how defensive symbionts in invasive pea aphids, *Acyrthosiphon pisum*, respond to emerging threats. Previously rare strains of the protective symbiont *Hamiltonella defensa* increased from <0.5% to 58% in aphid populations over just a few years. Bioassays revealed that these strains confer reciprocal, enemy-specific defences. One strain (C11) protected against *Praon pequodorum*, a native parasitoid that only began attacking pea aphids post-introduction, but provided no defence against the co-evolved parasitoid *Aphidius ervi.* Conversely, a closely related strain (C9) protected strongly against *A. ervi* but not *P. pequodorum.* When the Acyrthosiphon pisum secondary endosymbiont (APSE) bacteriophage was spontaneously lost from *H. defensa* C11 during cultivation, protection against *P. pequodorum* was completely eliminated, experimentally confirming the essential role of phage-encoded defences. Cultivation-assisted genomic analyses implicate divergent phage virulence cassettes in enemy-targeted defence, creating complementary protection portfolios within populations. The modular architecture of APSE phages enables rapid acquisition of novel capabilities through horizontal gene transfer. Critically, both strains maintained robust anti-parasitoid defence under simulated heat-wave conditions, in contrast to previous findings in which modest temperature increases disabled protection in other *H. defensa* strains. Our findings demonstrate the potential for heritable symbionts to provide rapid adaptive responses to anthropogenic stressors within ecological timescales, representing a widespread mechanism for host persistence under accelerating global change and having important implications for biological control and ecosystem management.

## Introduction

Rising temperatures and invasive species pose compounding threats to the world’s insects [1–3]. Climate-associated heat waves are increasing in frequency, severity, and duration, emerging as major threats to animal life and ecosystem health [4]. Simultaneously, biological invasions create novel ecological interactions that may act synergistically with climate stressors, further threatening organismal health and ecosystem stability [5–7]. Understanding how organisms respond to these compounding threats is crucial for predicting ecosystem responses. This is, in turn, vital to conservation in natural systems and sustainable pest management in agriculture.

The complexity of these responses is amplified by microbial symbionts, which are ubiquitous across eukaryotic life and often mediate critical interactions involved in resource acquisition and host defence [8, 9]. Symbiotic partnerships appear especially vulnerable to climate change. The most conspicuous examples involve coral bleaching, where warming oceans transform mutualistic partnerships into parasitism [10]. Heat waves can have widespread impacts on marine symbioses [11] with the potential for cascading ecosystem effects [12]. However, symbionts may also confer climate resilience through host-level phenotypes that enable tolerance to novel stresses, demonstrated by bleaching-resistant corals possessing thermotolerant algal symbionts [13].

Arthropods comprise the bulk of animal diversity in terrestrial systems and provide critical ecological services while also serving as major crop pests and disease vectors [14]. Despite their ecological importance, the effects of climate change on arthropod symbioses remain understudied [15, 16]. The thermal resilience of arthropod–microbe symbioses varies depending on transmission mode and evolutionary history. In obligate heritable symbioses, host restriction and transmission bottlenecks drive massive genome reduction in symbionts, including the loss of heat shock proteins that stabilize cellular components under thermal stress [17]. These processes are predicted to generally limit their host’s thermal tolerance, although other mechanisms of climate adaptation may occur. In contrast, environmentally acquired symbionts typically maintain large, flexible genomes and colonize diverse host species, potentially making them more resilient to environmental variation, including rising temperatures [18].

Heritable facultative symbionts occupy a middle ground between obligate and environmentally acquired symbioses, possessing intermediate-sized yet evolutionarily dynamic genomes [19]. These symbionts inhabit most insect species and frequently mediate ecological interactions with specialized natural enemies, including parasitoids and pathogens [20, 21]. The effects of rising temperatures and heat waves on facultative symbionts are predicted to be highly variable—some may increase host vulnerability to thermal stress, whereas others potentially provide thermal resilience, depending on their specific metabolic capabilities and stress response mechanisms [15–17, 22].

Aphids provide an ideal system for examining these interactions [23, 24]. These globally important phloem-feeding pests have colonized most agroecosystems [25]. They carry nine common heritable facultative symbionts, most of which provide defence against pathogens and parasites [24, 26–28]. The most common facultative symbiont, *Hamiltonella defensa*, protects against parasitoid attack through toxins encoded by the bacteriophage APSE [24, 29–33].

The North American pea aphid (*Acyrthosiphon pisum*) and parasitoid complex on alfalfa (*Medicago sativa*) exemplifies these interactions. The pea aphid was introduced in the nineteenth century and was initially attacked by various parasitoids until the intentional introduction of *Aphidius ervi* in 1959 [34]. *Aphidus ervi* became the dominant parasitoid, displacing most competitors except the native *Praon pequodorum*. Although *A. ervi* is the superior external competitor, *P. pequodorum* is the superior internal competitor during multiparasitism [35]. Our focal symbiont, *H. defensa*, exhibits substantial strain-level diversity, with isolates falling into five bacterial clades (A–E) and at least seven APSE phage types, each encoding distinct toxin cassettes [36–38]. All previously tested APSE-bearing isolates (spanning all bacterial clades and most APSE types) confer at least some protection against *A. ervi*, yet none have been found to defend against the native *P. pequodorum*. This leaves a reservoir of aphids susceptible only to the native parasitoid, which likely contributes to its persistence after the introduction of *A. ervi* [35].

The *H. defensa* protective symbiosis is also highly temperature sensitive—a mere 2.5°C increase, well within predicted climate warming ranges, partially or wholly disables protection against *A. ervi* across multiple *H. defensa* strains [39, 40]. Thus, pea aphids in North American alfalfa face a dual vulnerability: a native parasitoid against which no known *H. defensa* strain provides protection and a co-evolved parasitoid whose suppression is undermined by rising temperatures. Here, we investigate whether recently emerged, previously rare C-clade *H. defensa* strains might address one or both vulnerabilities using an integrated approach combining field surveys, genomics, and experimental bioassays. We find that C-clade isolates, which differ primarily in their APSE phage-encoded toxin cassettes, confer reciprocal, enemy-specific protection: one strain protects against *A. ervi* but not *P. pequodorum*, whereas the other protects against *P. pequodorum* but not *A. ervi.* Both strains provide thermally stable protection against both parasitoid species under simulated heat-wave conditions. These findings suggest that *H. defensa* possesses sufficient genetic diversity to rapidly evolve defences against the increasingly common dual challenges of biological invasions and climate change.

## Materials and methods

### Study organisms

The pea aphid, *A. pisum*, is a phloem-feeding pest of leguminous crops that reproduces via cyclical parthenogenesis but can be maintained as genetically identical clonal lines under long-day conditions (16L:8D). Experimental clonal lines were established by selecting individual females and maintaining them on broad bean plants (*Vicia faba* ‘Broad Windsor’) in controlled-environment chambers at 19 to 20°C.

*Aphidius ervi* and *P. pequodorum* (Hymenoptera: Braconidae: Aphidiinae) are the primary parasitoids of pea aphids on North American *M. sativa* [34]. Both are solitary endoparasitoids that oviposit in second- or third-instar nymphs, with larvae developing internally for ~1 week before killing the host and pupating within the desiccated exoskeleton (‘mummy’). Each species produces morphologically distinct mummies, enabling reliable identification. Parasitoid colonies were established from pea aphid mummies collected in Dane County, Wisconsin, and maintained at 20 ± 1°C under a 16L:8D photoperiod. Colonies were reared on aphid clones lacking anti-parasitoid symbionts or endogenous resistance [41]. Adult wasps were provided with honey and water.

### Pea aphid symbiont surveys

Pea aphids were collected from alfalfa fields near Arlington, WI (sampling individuals ~10 m apart to avoid clones) [42]. Collections occurred late May to early June 2021–2023 when pea aphids first became abundant, as in our earlier 2014–2019 study [36]. DNA was extracted from field-collected individuals using the E.Z.N.A. Tissue DNA Kit (Omega Biotek) and stored at −80°C. V3f/V4r 16S rRNA gene regions of pooled samples sequenced on a MiSeq System (Illumina) revealed only seven previously reported facultative symbionts (*Hamiltonella*, *Rickettsia*, *Fukatsuia*, *Rickettsiella*, *Regiella*, *Serratia*, and *Spiroplasma*) [36]; we therefore screened all individuals for these symbionts using established primers that are diagnostic for each [42].

For *H. defensa*–positive aphids, we characterized bacterial clades using five loci (*ptsI*, *recJ*, *accD*, *murE*, and *hrpA*) and APSE variants using four markers (P3, P35, P38, and P45) following previously published methods [36, 43]. Simpson diversity index calculations were conducted using vegan: Community Ecology Package 2.7-1 (10.32614/CRAN.package.vegan), and differences in C-strain frequency between time periods were tested using Fisher’s exact test. Both were performed using R Statistical Software (v4.5.1; R Core Team, 2025m, Vienna, https://www.R-project.org/) with the RStudio interface (Posit team, 2025, Boston http://www.posit.co/).

### Creation of experimental lines

We transferred focal C-clade *H. defensa* isolates (Table 1) into two parasitoid-susceptible aphid genotypes (5D and ND18; Table 1) via microinjection following established protocols [44]. Strains 3492 and 2185 initially harboured APSE11, a recently discovered phage variant [37]. Spontaneous phage loss of APSE11 from line 2185 (denoted 2185x) enabled direct assessment of APSE’s defensive role. Aphids were reared as described above and re-screened for symbionts and phage immediately before experimental assays.

**Table 1:** Experimental *Acyrthosiphon pisum* lines used in this study.

*H. defensa* isolate aphid clone	*H. defensa* collection info	Aphid clone collection info	*H. defensa* clade	APSE variant and toxin gene	Creation method
5D		5D (WI, 2012)	None	None	AB
3492 → 5D	WI, 2018		C	APSE11 (*ydp2*)	Micro
2185 → 5D	WI, 2018				
2185x → 5D	WI, 2018			No APSE	VTF
ND18		ND18 (ND, 2018)	None	None	Natural
4721 → ND18	WI, 2020		C	APSE9 (*cdtB*_3_)	Micro
3483 → ND18	WI, 2020				
I928 → ND18	NY, 2020				

All aphids were collected on alfalfa (*Medicago sativa*) and clonal lines were established from single parthenogenetic females. MI, Michigan; PA, Pennsylvania; WI, Wisconsin; ND, North Dakota (all locales in USA); AB, antibiotic cured; Micro, microinjection; VTF, vertical transmission failure.

### Culture-assisted genomics

We cultured *H. defensa* C11 isolates 3492 and 2185 (Table 1) and C9 isolate I928 to generate DNA templates free of aphid or *Buchnera* contamination [45–47]. High-quality long-read genomes were generated using PacBio Sequel II Single Molecule Real-Time (SMRT) sequencing technology, which is critical for comparative genomics of strains that vary primarily in mobile or repetitive DNA elements [48]. Combined with the previously sequenced C9 isolate MI12 [37], this approach provided four C-clade isolates for comprehensive comparative analysis. Sequencing performed at the Georgia Genomics and Bioinformatics Core yielded >100 million corrected base pairs and ~6600 reads per genome, with N50 lengths of 11 609 to 15 812 bp (Table S1). Genome assemblies were constructed using Canu v2.2 [49].

Coding sequences were predicted using two complementary approaches: NCBI’s Prokaryotic Genome Annotation Pipeline (PGAP) [50] and the RAST Toolkit (RASTtk) [51]. Predictions from both pipelines were subsequently integrated using Geneious Prime v2025.2.2 (www.geneious.com). Homopolymer-associated insertion–deletion mutations were manually corrected during assembly refinement. Sequences exhibiting <80% completeness relative to reference sequences were designated pseudogenes following established criteria [46].

Ribosomal RNA gene identification was performed using RNAmmer v1.2 and tRNAscan-SE v2.0 was used for transfer RNA genes [52, 53]. Mobile genetic elements including prophages and insertion sequences were characterized using PHASTER and ISEScan [54, 55]. Genome alignments, comparative analyses, average nucleotide identity (ANI), and average amino acid identity calculations were performed and visualized using progressiveMauve v2.4.0 and Proksee [56, 57]. Coding domain sequences (CDSs) from the C9 and C11 *H. defensa* strains were compared using BLASTp searches [58] against a local database containing both genomes. BLAST v2.12.0+ was employed with default parameters, followed by manual curation of results.

We inferred a phylogeny placing our focal C-clade isolates in the context of publicly available complete *H. defensa* genomes, based on single-copy core orthologous (SICO) genes identified using Roary (v3.11.2) [59]. In total, 304 core genes present as a single copy across all genomes were extracted and concatenated (Table S2), and the resulting alignment was used to infer a maximum-likelihood phylogeny in IQ-TREE (v2.2.2.6) with the best-fit substitution model and 10 000 bootstrap replicates [60]. The final tree was visualized and annotated in Geneious Prime v2025.2.2 (https://www.geneious.com). Table S3 lists genome project and assembly accession numbers for genomes used in the phylogeny.

### Parasitism assays

We conducted two experimental series. First, ‘standard’ assays at constant 20°C investigated whether C9 and C11 isolates protect against *A. ervi* and *P. pequodorum*, enabling comparison with previous studies. Second, thermal regime assays evaluated symbiont defensive services under contrasting conditions: ‘cool’ (daily mean 18°C, cycling 14.6°C at 6:00 a.m. to 22°C at 7:00 p.m.) and ‘heat wave’ (daily mean 25°C, cycling 19°C at 6:00 a.m. to 32°C at 7:00 p.m.). These regimens simulated heat waves with mean temperatures +7°C above the ‘cool’ conditions, which represent typical early June temperatures at our collection sites.

Individual second- or third-instar nymphs were singly parasitized by mated female wasps and placed in groups of 20 on potted fava bean seedlings within 16-ounce mesh-lidded cages. Cages were randomly assigned to thermal treatments under a 16L:8D photoperiod. After 10 days, we recorded three mutually exclusive outcomes: surviving aphids (AA), mummies representing successful wasp development (M)—an established proxy for successful parasitism [61], and dual mortality (DM). All parasitism assays were conducted at least five generations after symbiont transfer to minimize residual effects from manipulations [62].

Data were analysed using generalized linear models with binomial distribution and logit link function, examining three contrasts: (i) aphid survival versus parasitoid success plus dual mortality (AA vs. M + DM), (ii) parasitoid success versus aphid survival plus dual mortality (M vs. AA + DM), and (iii) dual mortality versus any survival (DM vs. AA + M). Firth bias-adjusted estimates addressed sparse data cells when needed (Table S4). Model fit was assessed using likelihood ratio tests and overdispersion evaluated using Pearson chi-square statistics. Analyses were performed in JMP Pro 17 and JMP Student Edition 18 (SAS Institute Inc.).

## Results

### Temporal shifts in *H. defensa* strain frequencies

**Figure 1:**
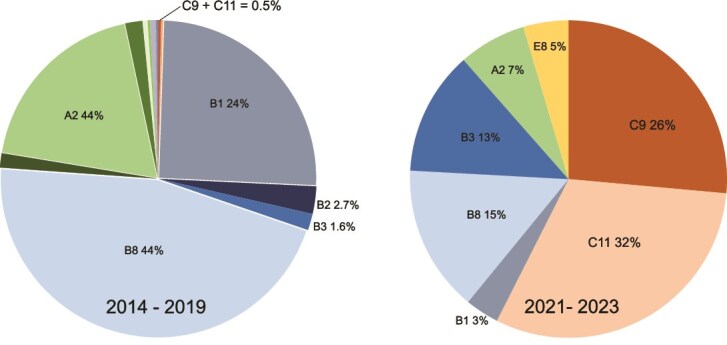
Pie charts representing the percentage of sampled aphids carrying different strains of the protective symbiont, *Hamiltonella defensa* from fields in Arlington, WI, USA*.* (A) *2014–2019* (*N* = 634) data extracted from [41], (B) 2021–2023 (=88) samples are new to this study. *N* = number of individual aphids with *H. defensa*.

Population surveys revealed striking temporal shifts in the frequencies *of H. defensa* strains at our Arlington, WI site. *Hamiltonella defensa* was the most common facultative symbiont, infecting 56% of pea aphids from 2014 to 2019 [36]. Prior to 2020, A- and B-clade strains predominated, with A2, B1, and B8 comprising 86% of sampled aphids (*N* = 634), whereas C-clade strains (C9 and C11) were rare at just 0.47% combined [36]. Collections were not made in 2020 due to COVID-19 disruptions, but smaller collections of *H. defensa*–carrying aphids (*N* = 88) from the same fields between 2021and 2023 revealed a large community shift. C-Clade strains increased 290-fold to comprise 58% of sampled aphids (Fisher’s exact test *P* value <.0001; odds ratio = 289.9, 95% CI: 86.4–972.9), with C9 and C11 being the only C-clade strains recovered (Fig. 1). Despite this large compositional change and fewer total strains detected in the recent sample, overall diversity remained similar between the two collection periods (Simpson’s diversity: 0.71 vs 0.79), indicating community turnover rather than diversity loss.

### *Hamiltonella defensa* strain emerges that targets a novel parasitoid

**Figure 2:**
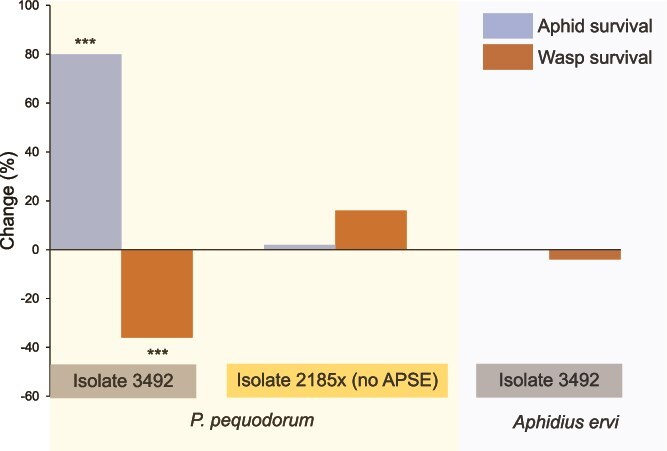
C11 *H. defensa* harms *P. pequodorum* but not *A. ervi.* Change in odds of aphid survival and wasp survival in aphids carrying C11 isolates of *H. defensa* compared to symbiont-free controls (all aphids are genotype 5D). *Hamiltonella defensa* isolates (e.g. 3492) and parasitoid species are noted below the columns. ^***^P value <.0001 from GzLM (Table S4A).

Standard temperature assays (20°C constant) revealed that C11 *H. defensa* isolate 3492 → 5D conferred significant protection against *P. pequodorum* compared to uninfected controls (5D). The overall model was significant (χ^2^ = 21.6, *df* = 4, *P* value *<* .0001, *N* = 462), with an 80% increase in aphid survival odds (χ^2^ = 16.2, *P* value < .0001; OR = 1.8 [95% CI: 1.33–2.28]) and 36% decrease in mummy production (χ^2^ = 15.4, *P* value < .0001; OR = 0.64 [95% CI: 0.51–0.81]) compared to symbiont-free controls (Table S4A; Fig. 2). Among the many *H. defensa* strains previously assayed for parasitoid protection in this introduced aphid [30], C11 is the first *H. defensa* found to protect against a native parasitoid that shifted to exploit the introduced host.

### Protection is parasitoid specific

**Figure 3:**
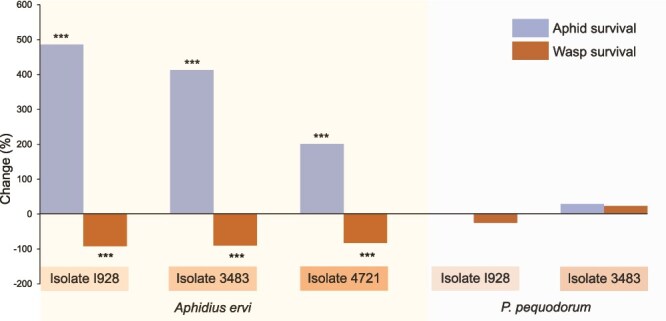
C9 *H. defensa* harms *A. ervi* but not *P. pequodorum.* Change in aphid survival and wasp survival in aphids carrying C9 isolates of *H. defensa* compared to symbiont-free controls (all aphids are genotype ND18). *Hamiltonella defensa* isolates (e.g. I928) and parasitoid species are noted below the columns. ^***^P value <.0001 from GzLM (Table S4B).

Despite protecting against *P. pequodorum*, C11 isolate 3492 → 5D provided no defence against *A. ervi*, with survival (χ^2^ = 0.003, *P* value = .96) and mummification (χ^2^ = 0.18, *P* value = .67) rates equivalent to symbiont-free controls (Fig. 2). Both parasitoid species successfully parasitized the uninfected 5D control at similar rates (χ^2^ = 0.01, *P* value = .93). Conversely, C9 *H. defensa* isolates provided strong protection against *A. ervi* but offered no protection against *P. pequodorum* in 20°C bioassays. Three C9 isolates significantly improved aphid survival against *A. ervi* (Fig. 3, Table S4B, all *P* values < .0001), with 201%–486% increased survival odds and 83%–92% reductions in mummification compared to symbiont-free controls. Two isolates tested against *P. pequodorum* showed neither improved aphid survival nor reduced wasp development (Fig. 3, Table S4). Parasitism success did not differ between the two parasitoid species attacking the ND18 control aphids (χ^2^ = 1.64, *P* value = .20).

### APSE toxin cassettes implicated in parasitoid-specific defence

To confirm the mechanistic basis of protection, we examined a second C11 isolate (2185x → 5D) that had spontaneously lost bacteriophage APSE11 during laboratory cultivation. This phage-cured strain completely lost protective capacity against *P. pequodorum*—aphid survival did not differ from symbiont-free controls (χ^2^ = 0.002, *P* value = .96), and mummy production was similar (χ^2^ = 1.29, *P* value = .26) (Fig. 2). This loss-of-function phenotype directly demonstrates that APSE11 is necessary for protection against *P. pequodorum*.

**Figure 4:**
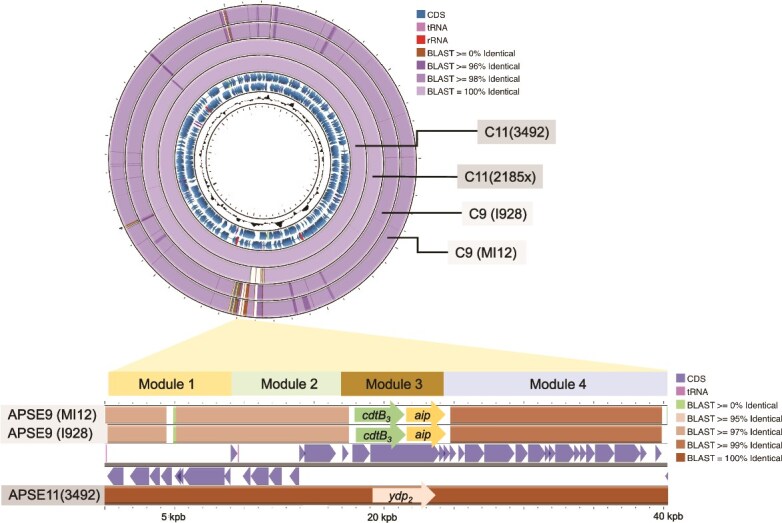
Comparative genomic analysis of C-clade *Hamiltonella defensa* strains. (top) Circular genome comparison of C-clade isolates mapped against the C11(3492) reference; rings from inside to outside represent C11(2185x), C9(I928), and C9(MI12) with colour intensity reflecting nucleotide identity. The two C11 isolates are nearly identical except that 2185x spontaneously lost bacteriophage APSE11 during laboratory cultivation. The two C9 are also nearly identical to each other and broadly similar to C11 strains but carry distinct APSE variants. (bottom) Linear comparison of the APSE genomic region illustrating modular phage organization (modules 1–4). APSE9 (C9 isolates) encodes a *cdtB3-aip* virulence cassette in module 3, whereas APSE11 (C11 isolates) encodes *ydp2*. Colour shading indicates nucleotide identity.

The critical role of APSE11 is further supported by comparative genomic analysis. Cultivation-assisted PacBio sequencing of C11 *H. defensa* isolates 3492 and 2185 revealed that they were nearly identical (Fig. 4; Table S5). The complete 2.12 Mb genomes of C11 isolates 3492 and 2185 shared nearly 100% ANI and genomic architecture: 40.7% GC content, ~2000 predicted coding sequences (CDSs), 43 tRNAs, and 9 rRNAs. Mobile genetic elements were also virtually identical between isolates, including 85 insertion sequences (IS elements), 16 prophage regions, 4 extracellular plasmids, and 17 plasmid-derived sequences. This high level of similarity strongly supports that the presence or absence of APSE11 alone accounts for the differential protective phenotypes.

Both C11 isolates originally harboured APSE11 with identical YDp2 toxin alleles. This YDp2 variant differs from the YDp1 allele found in D-clade *H. defensa* strains carrying APSE3, which confer strong protection against *A. ervi* but not against *P. pequodorum* [37, 44]. This allelic variation suggests that toxin sequence determines parasitoid host specificity.

C9 isolates I928 and MI12 were likewise nearly identical (ANI 99.99%), including identical *cdtB3*-*aip* toxin duplexes (Figs. 4 and S1), and were highly similar to C11 isolates (ANI = 99.81 and 99.83%; Fig. S1). The principal difference between C9 and C11 resides in their APSE content: C9 harboured *cdtB3*-*aip*-encoding APSE9, whereas C11 carried *ydp2*-encoding APSE11 (Fig. 4). Beyond the phage differences, the strains showed minor genomic variation: C9 isolate I928 contained an extrachromosomal plasmid (pHDI928.p1) not found in the C11 isolate, whereas C11 harboured a unique extrachromosomal plasmid (pHD3492.1) along with several additional coding sequences absent from C9. However, homology searches indicate that none of these additional loci are predicted to influence parasitism outcomes, and all are present in other *H. defensa* strains (Table S5).

The experimental loss-of-function data and comparative genomics identify APSE as the critical determinant of protection: APSE11 is necessary for defence against *P. pequodorum*, the C9 and C11 strains differ primarily in their APSE content, and their respective toxin cassettes co-vary precisely with the observed parasitoid-specific protection phenotypes.

### C-Clade *H. defensa* symbionts provide robust defence at warmer temperatures

We tested whether C-clade strains retain their defensive effectiveness under warmer temperature regimens that simulated heat waves. Symbiont-free control aphids (5D) showed no difference in parasitism success between cool and heat-wave conditions for either parasitoid (*P. pequodorum*: χ^2^ = 0.69, *P* value = .41; *A. ervi*: χ^2^ = 0.53, *P* value = .47), confirming that temperature alone does not affect parasitism in this aphid genotype.

**Figure 5:**
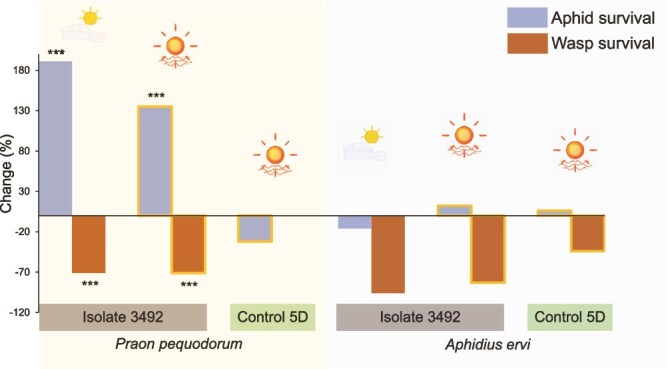
C11 *H. defensa* remains robust to *P. pequodorum* parasitism during simulated heat wave. Change in odds of aphid and wasp survival in aphids carrying C11 *H. defensa* at cool (indicated by partly cloudy icon) and heat-wave (sun icon) temperature regimens compared to symbiont-free controls at cool temperatures. All aphids are genotype 5D. *Hamiltonella defensa* isolates (e.g. 3492) and parasitoid species are noted below the columns. ^***^P value <.0001 from GzLM (Table S4C).

The C11 *H. defensa* isolate (3492 → 5D) decreased *P. pequodorum* mummification odds by 71% under both cool (χ^2^ = 43.5, *P* value < .0001) and heat-wave conditions (χ^2^ = 53.7, *P* value < .0001), and significantly increased aphid survival by 191% (cool) and 135% (heat wave) compared to controls (Fig. 5, Table S4). This demonstrates that C11 symbiont protection against *P. pequodorum* is thermally robust.

Because previously characterized *H. defensa* strains protect against *A. ervi*, we also tested whether C11’s failure to protect against this parasitoid at standard temperatures might represent a thermally conditional phenotype, specifically, whether protection could emerge at warmer temperatures. The C11 isolate provided no protection against *A. ervi* under either cool (χ^2^ = 0.35, *P* value = .56) or heat-wave conditions (χ^2^ = 0.07, *P* value = .78) (Fig. 5), confirming that C11’s specificity for *P. pequodorum* is maintained regardless of temperature.

**Figure 6:**
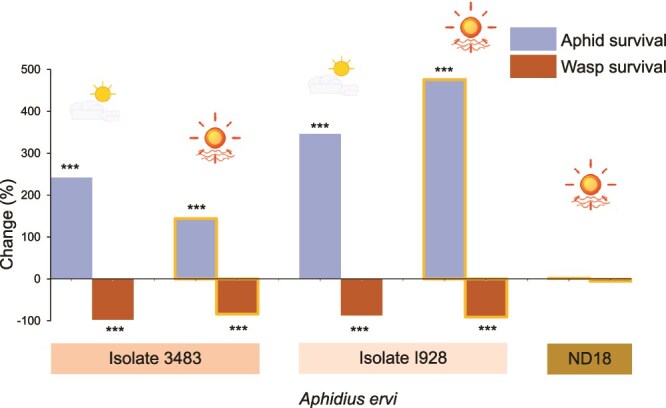
C9 *H. defensa* remains robust to *A. ervi* parasitism during simulated heat wave. Change in odds of aphid and wasp survival in aphids carrying C9 *H. defensa* at cool (indicated by partly cloudy icon) and heat-wave (sun icon) temperature regimes compared to symbiont-free controls at cool temperatures. All aphids are genotype ND18. *Hamiltonella defensa* isolates (e.g. 3482) are noted below the columns. Only *Aphidius ervi* was used in this assay. ^***^P value <.0001 from GzLM (Table S4D).

To determine whether C9 *H. defensa* protection against *A. ervi* is thermally robust, we compared parasitism outcomes under cool and heat-wave conditions. Parasitism success did not differ between cool and heat-wave conditions for the symbiont-free control line ND18 (χ^2^ = 0.06, *P* value = .81), indicating that temperature alone does not impact *A. ervi* parasitism in this genotype. In contrast, two C9 isolates decreased *A. ervi* mummification odds by 87% (I928; χ^2^ = 224.4, *P* value < .0001) and 97.5% (3483; χ^2^ = 553.7, *P* value < .0001) at cool temperatures, and by 91% (I928; χ^2^ = 263.0, *P* value < .0001) and 84% (3483; χ^2^ = 215.6, *P* value < .0001) during simulated heat waves compared to symbiont-free controls (Fig. 6). Similarly, aphid survival was greatly increased for both C9 isolates (346% for I928, 367% for 3483; both *P* values < .0001, Table S4). For isolate I928, the C9 *H. defensa* enhanced protection during heat waves compared with cool conditions, with improved aphid survival and decreased mummification.

## Discussion

### Target-specific protection against a native parasitoid in an introduced pest

The C11 *H. defensa* strains present in introduced pea aphids represent the first documented case of protection against *P. pequodorum*, a native parasitoid that underwent host switching to exploit this aphid following its nineteenth-century arrival in North America. This protective specificity contrasts sharply with the well-characterized strain diversity of *H. defensa* in North American alfalfa populations, where extensive studies have shown that all tested isolates from other *H. defensa* clades and APSE types provide protection exclusively against the co-evolved parasitoid *A. ervi*, conferring no resistance to *P. pequodorum* [30, 35, 63, 64].

Developmental differences between these two parasitoids, including *P. pequodorum*’s prolonged development within a more heavily chorionated egg, were previously hypothesized to explain why multiple *H. defensa* strains failed to harm *P. pequodorum* [64]. The ability of C11 strains to overcome *P. pequodorum* suggests that symbiont factors from this strain can penetrate the parasitoid’s enhanced defences, though further investigation is needed to determine the precise timing and mechanism of *P. pequodorum* mortality in C11-defended aphids.

C11 *H. defensa* exhibited strict target specificity, protecting against *P. pequodorum* but providing no defence against *A. ervi* (Fig. 2). Outside of *H. defensa* isolates that lack APSE phage [29, 65], this represents the first pea aphid *H. defensa* strain sampled in North America documented to fail against *A. ervi* [30], suggesting evolutionary trade-offs in defensive capabilities between parasitoid species.

Closely related C9 *H. defensa* isolates provided robust protection against *A. ervi* but offered no defence against *P. pequodorum* (Fig. 3), reinforcing that defensive mechanisms in these symbionts are highly targeted rather than broad spectrum. The coexistence of C9 and C11 strains within aphid populations creates complementary protection against distinct parasitoid threats, potentially representing an adaptive portfolio response to selection pressures from multiple natural enemies. Pea aphids also encode some endogenous resistance to *A. ervi* [66], but none has been reported against *P. pequodorum*, consistent with symbiont protection evolving faster than host-encoded defences.

Target specificity of *H. defensa* has been documented in other aphid species [67–70] and in *Spiroplasma*, another facultative symbiont that protects aphids against parasitoids [71], suggesting that specialized rather than generalized defensive strategies may be the norm for many protective symbioses. In the black bean aphid, *Aphis fabae*, target specificity occurs among *H. defensa* strains and parasitoid genotypes [72], indicating that this specificity can operate across multiple levels.

### Enhanced thermal tolerance

Both C9 and C11 *H. defensa* strains demonstrated thermally robust anti-parasitoid defences under simulated heat-wave conditions (Figs. 5 and 6), contrasting with previous findings that even modest increases of 2.5°C above average temperatures disabled protection in other *H. defensa* strains [39, 73]. Our results show that defensive effectiveness is maintained across thermal treatments, with some evidence of enhanced protection under heat stress for one C9 isolate (Fig. 6). This symbiont-mediated thermal robustness to parasitism represents a potentially critical adaptation for aphids facing increasingly frequent and severe heat events [4]. The mechanistic basis for this thermally stable protection remains unclear and requires further study.

Thermally robust symbiont-mediated defence builds on a foundation of prior work showing that temperature profoundly affects defensive symbioses by altering transmission rates, symbiont titres, and phenotypes [22, 74–79]. Beyond these impacts on defensive function, previous research has identified thermal tolerance benefits conferred by aphid facultative symbionts in the absence of enemy challenge. Specifically, *Serratia symbiotica*, *Fukatsuia symbiotica*, and certain *H. defensa* strains improve aphid fitness during heat shocks—short bursts of extreme heat—possibly by compensating for thermosensitive obligate symbionts [64, 76–78]. Whether these protective mechanisms extend to the sustained elevated temperatures characteristic of ecologically realistic heat waves remains unclear. If so, thermal tolerance would represent a complementary avenue for symbiont-mediated adaptation to climate change, operating alongside the maintenance of enemy defence demonstrated here.

### Standing genetic variation potentially enables rapid symbiont-mediated adaptation

Our strain-level surveys of *H. defensa* in Wisconsin pea aphid populations revealed that C-clade *H. defensa* strains that were each less than 0.3% of those sampled between 2014 and 2019 [36] comprised nearly 60% of the sampled population from 2021 to 2023 (Fig. 1). This represents a large shift in the defensive symbiont landscape. We note that this comparison spans a single pair of time periods with unequal sample sizes, and that drift or abiotic factors cannot be excluded without more extensive temporal sampling. Nevertheless, the protective phenotypes we identified in these strains, defence against a novel parasitoid and thermally stable protection against parasitoids, provide plausible mechanisms that could drive such rapid changes in symbiont composition. This demonstrates that *H. defensa* harbours the genetic variation necessary to respond to novel environmental challenges and illustrates the potential for rapid symbiont-mediated adaptation to emerging threats, though field validation of these selective mechanisms remains an important next step.

### Bacteriophage-mediated resistance and specificity

The essential role of APSE bacteriophages in *H. defensa* protection was experimentally confirmed by analysing C11 isolates that carry APSE11 with YDp2 toxins. When C11 isolate 2185 spontaneously lost its APSE11 phage (designated 2185x), it completely lost protective capacity against *P. pequodorum* (Fig. 2). Comparative genomic analysis revealed that protective (3492) and non-protective (2185x) isolates were virtually identical except for APSE11 presence, providing only the second experimental demonstration that APSE bacteriophages are essential for anti-parasitoid protection [29].

Prior genomic studies indicate that within-clade *H. defensa* isolates share highly similar genome content and organization, including mobile genetic element composition, whereas isolates from distinct clades differ substantially in these features. Within clades, however, isolates can vary in APSE type, and this variation correlates with differences in the strength of protection against the co-evolved *A. ervi* [37]. Here, we show that C-clade *H. defensa* isolates, differing primarily in their toxin-encoding virulence cassettes, target distinct parasitoid species: C9 isolates harbour *cdtB_3_-aip*-encoding APSE9 that protect against *A. ervi*, whereas C11 isolates carry ydp2-encoding APSE11 that defend against *P. pequodorum*. The conservation of bacterial chromosomes and APSE backbone sequences, including phage regulatory elements, across these strains makes variation in the toxin cassettes themselves the most parsimonious explanation for target specificity, though definitively resolving whether specificity reflects toxin sequence, regulatory differences, or both would require functional characterization beyond the scope of this study. Beyond differences in APSE presence and type, the only other major genomic variation observed was in plasmid number and identity (the C9 isolate carried 2 plasmids, whereas C11 isolates carried 4 or 5). However, analysis of plasmid gene inventories and their inconsistent presence across strains with anti-parasitoid function indicates that plasmids are unlikely to contribute to the observed target specificity, though they may serve other roles in the symbiosis.

The diversity of *H. defensa*/APSE/toxin combinations in Wisconsin alfalfa populations [36], where toxins move among phage backbones and phage among *H. defensa*, illustrates the potential for horizontal gene transfer of bacteriophage to rapidly generate novel defensive phenotypes within species, allowing symbionts to evolve targeted responses against different enemy species through modular acquisition of specialized molecular weaponry. A recent study of European aphid species independently identified APSE toxin module variation as the principal driver of target-specific protection, strengthening the generality of this conclusion across diverse aphid–parasitoid systems [80].

## Conclusions

The emergence of C-clade *H. defensa* strains with novel defensive targets and thermally robust defensive capabilities demonstrates that heritable symbionts have the potential to provide rapid, adaptive responses to anthropogenic stressors, including climate change and species interactions resulting from the introduction of non-native species. In our case, a newly emerged symbiont strain enabled an invasive aphid to defend itself against a native parasitoid, illustrating how symbionts may facilitate biological invasions by creating additional advantages in introduced environments. This work highlights the importance of strain-level variation among insect microbial partners because both parasitoid targeting and thermal robustness were determined by specific strains.

The bacteriophage-mediated basis of these strain-specific defences provides a mechanism for rapid adaptation via symbiosis. Just as phages in bacterial pathogens are sources of evolutionary novelty [81], APSE phages in *H. defensa* encode target-specific virulence cassettes that confer anti-parasitoid protection to aphid hosts. The modular nature of this system, involving recombination within and among phages, horizontal transfer of APSE elements among symbionts, and lateral movement of symbionts among host species, can rapidly generate and disseminate new protective phenotypes within ecological timescales [29, 37, 43, 80, 82]. Conversely, phage loss eliminates protection and imposes fitness costs that may drive symbiont elimination [83], demonstrating the dynamic nature of symbiont-mediated adaptation and its potential to buffer host populations in changing environments.

Rapid symbiont-mediated adaptation may propel eco-evolutionary dynamics, where evolutionary changes occur fast enough to influence contemporary ecological processes and community interactions [84]. The modular nature of APSE-*Hamiltonella* may enable rapid defence evolution, allowing for feedback loops that alter species interactions and, in turn, select for further symbiont evolution. Combined with the costs of defence (i.e. trade-offs) and constraints imposed by dispersal patterns [85, 86], this dynamic interplay between symbiont evolution and ecological processes may be essential for determining community composition, stability, and persistence in changing environments.

Our findings have practical implications for biological control programmes, which increasingly operate under changing climatic conditions and novel species assemblages [87, 88]. The rapid evolutionary potential of these symbioses indicates that biological control outcomes may shift over relatively short time periods as symbiont communities respond to selection pressures.

Microbial symbionts represent an underappreciated source of adaptive capacity in anthropogenic environments. As global change accelerates, understanding and potentially harnessing symbiont-mediated adaptation may prove necessary for both conservation efforts and sustainable management of agricultural systems.

## Supplementary Material

Figure_S1_updated_wrag098

supplemental_revision_Apr14_wrag098

## Data Availability

Whole-genome sequences generated in this study have been deposited in the NCBI database under accessions CP133792–CP133796, CP133493–CP133498, and CP133801–CP133803 for *H. defensa* isolates 3492, 2185, and I928, respectively. All parasitism data are available on figshare at https://doi.org/10.6084/m9.figshare.31935663.
