# Supplementary material for: Emergent symbiont strains provide thermally robust protection against co-evolved and novel parasitoids of introduced pea aphids
Source: ISME J. 2026 Apr 17;20(1):wrag098. doi: 10.1093/ismejo/wrag098 (PMC13184529; doi:10.1093/ismejo/wrag098)
Supplement: Figure_S1_updated_wrag098 [file figure_s1_updated_wrag098.pdf]

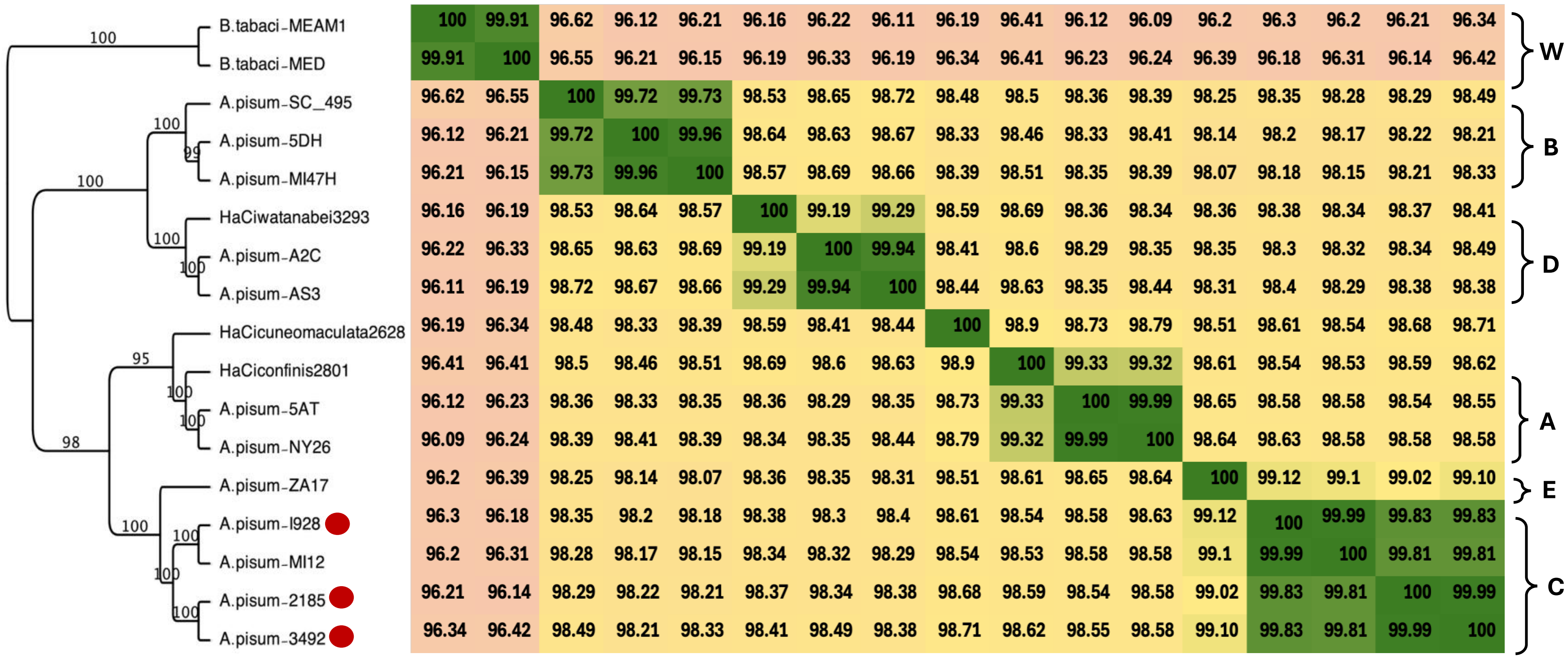

Figure S1. Maximum likelihood phylogeny and average nucleotide identity (ANI) of *H. defensa* isolates. The phylogeny was inferred from a concatenated alignment of 304 single-copy core orthologous genes (Table S2) identified across all publicly available complete *H. defensa* genomes using Roary (v3.11.2), with the three focal isolates from this study indicated by filled red circles. The maximum likelihood tree was constructed in IQ-TREE (v2.2.2.6) using the best-fit substitution model (GTR+F+I+G4) with 10,000 bootstrap replicates; bootstrap support values are shown at nodes. Clades are designated following Patel et al. (2023); whitefly-associated *H. defensa* strains are grouped as the W clade. The heatmap displays pairwise ANI values (%) for all genome pairs, with green indicating higher identity. NCBI BioProject and assembly accession numbers for all genomes are provided in Table S3.
