# Supplementary material for: Emergent symbiont strains provide thermally robust protection against co-evolved and novel parasitoids of introduced pea aphids
Source: ISME J. 2026 Apr 17;20(1):wrag098. doi: 10.1093/ismejo/wrag098 (PMC13184529; doi:10.1093/ismejo/wrag098)
Supplement: supplemental_revision_Apr14_wrag098 [file supplemental_revision_apr14_wrag098.pdf]

Supplementary tables for "Emergent symbiont strains provide thermally robust protection against co-evolved and novel parasitoids of introduced pea aphids"

Table of contents

Table S1: Summary of PacBio sequencing information (pg. 1)
Table S2: The 304 core genes used to infer phylogeny (pg. 2)
Table S3: Accession numbers for genomes used to produce phylogeny (pg. 9)
Table S4: Generalized linear model results for parasitism assays (pg. 10)
Table S5: CDS differences among C-clade *H. defensa* strains (pg. 15)

Table S1: Summary of PacBio sequencing information

***H. defensa* C-clade isolates**	**3492**	**2185**	**I928**
Original reads (bp)	48,932	48,039	46,661
Original bases	500,007,163	500,002,099	500,002,965
Corrected reads (bp)	6,552	6,685	6,613
Corrected bases	102,620,601	102,409,808	103,929096
Maximum read length (bp)	28,131	32,823	33,215
N50	15,689	15,812	11,609
Coverage (X)	41.04	40.89	41.57
**Genomic information**			
Chromosome Size (Mb)	2.123	2.122	2.123
Plasmid	4	5	2
Coding Sequences (CDS)	2130	2049	2051
CDS on Plasmid	230	235	107
rRNA	9	9	9
tRNA	43	43	42
APSE variant	APSE11	APSE11	APSE9

Table S2: The 304 core genes used to infer phylogeny.

Gene	Annotation
accA	Acetyl-coenzyme A carboxylase carboxyl transferase subunit alpha
accB	Biotin carboxyl carrier protein of acetyl-CoA carboxylase
accC	Biotin carboxylase
accD	Acetyl-coenzyme A carboxylase carboxyl transferase subunit beta
aceE	Pyruvate dehydrogenase E1 component
aceF	Dihydrolipoyllysine-residue acetyltransferase component of pyruvate dehydrogenase complex
ackA	Acetate kinase
ahpC	Alkyl hydroperoxide reductase C
arnA	Bifunctional polymyxin resistance protein ArnA
arnT_2	Undecaprenyl phosphate-alpha-4-amino-4-deoxy-L-arabinose arabinosyl transferase
artM	Arginine ABC transporter permease protein ArtM
artP	Arginine transport ATP-binding protein ArtP
asnB	Asparagine synthetase B [glutamine-hydrolyzing]
aspC	Aspartate aminotransferase
aspS	Aspartate--tRNA ligase
atpA	ATP synthase subunit alpha
atpB	ATP synthase subunit a
atpD	ATP synthase subunit beta
atpE	ATP synthase subunit c
atpF	ATP synthase subunit b
atpG	ATP synthase gamma chain
can	Carbonic anhydrase 2
carB	Carbamoyl-phosphate synthase large chain
cdsA	Phosphatidate cytidylyltransferase
clpP	ATP-dependent Clp protease proteolytic subunit
clpX	ATP-dependent Clp protease ATP-binding subunit ClpX
clsA	Cardiolipin synthase A
coaA	Pantothenate kinase
corC	Magnesium and cobalt efflux protein CorC
crr	PTS system glucose-specific EIIA component
cspE_2	Cold shock-like protein CspE
cvrA	K(+)/H(+) antiporter NhaP2
cyoA	Cytochrome bo(3) ubiquinol oxidase subunit 2
dam	DNA adenine methylase
dapD	2,3,4,5-tetrahydropyridine-2,6-dicarboxylate N-succinyltransferase
dapE	Succinyl-diaminopimelate desuccinylase
dapF	Diaminopimelate epimerase
dapH	2,3,4,5-tetrahydropyridine-2,6-dicarboxylate N-acetyltransferase
dcd	dCTP deaminase
deaD	ATP-dependent RNA helicase DeaD
degP	Periplasmic serine endoprotease DegP
djlA	Co-chaperone protein DjlA
dksA	RNA polymerase-binding transcription factor DksA
dnaA	Chromosomal replication initiator protein DnaA

dnaB	Replicative DNA helicase
dnaE	DNA polymerase III subunit alpha
dnaJ	Chaperone protein DnaJ
dnaN	Beta sliding clamp
dsbA	Thiol:disulfide interchange protein DsbA
dut	Deoxyuridine 5'-triphosphate nucleotidohydrolase
dxr	1-deoxy-D-xylulose 5-phosphate reductoisomerase
dxs	1-deoxy-D-xylulose-5-phosphate synthase
efp	Elongation factor P
epmA	Elongation factor P--(R)-beta-lysine ligase
erpA	Iron-sulfur cluster insertion protein ErpA
fabA	3-hydroxydecanoyl-[acyl-carrier-protein] dehydratase
fabB	3-oxoacyl-[acyl-carrier-protein] synthase 1
fabD	Malonyl CoA-acyl carrier protein transacylase
fabG	3-oxoacyl-[acyl-carrier-protein] reductase FabG
fabH	3-oxoacyl-[acyl-carrier-protein] synthase 3
fabZ	3-hydroxyacyl-[acyl-carrier-protein] dehydratase FabZ
fbaA	Fructose-bisphosphate aldolase class 2
feoC	putative [Fe-S]-dependent transcriptional repressor FeoC
folD	Bifunctional protein FolD protein
folE	GTP cyclohydrolase 1
fpr	Flavodoxin/ferredoxin--NADP reductase
fre	NAD(P)H-flavin reductase
ftsA	Cell division protein FtsA
ftsH	ATP-dependent zinc metalloprotease FtsH
ftsY	Signal recognition particle receptor FtsY
ftsZ	Cell division protein FtsZ
fumC	Fumarate hydratase class II
fur	Ferric uptake regulation protein
fusA	Elongation factor G
galU	UTP--glucose-1-phosphate uridylyltransferase
gapA	Glyceraldehyde-3-phosphate dehydrogenase A
glnP	Glutamine transport system permease protein GlnP
glnQ_1	Glutamine transport ATP-binding protein GlnQ
glnQ_2	Glutamine transport ATP-binding protein GlnQ
gltA	Citrate synthase
glyQ	Glycine--tRNA ligase alpha subunit
glyS	Glycine--tRNA ligase beta subunit
gmhA	Phosphoheptose isomerase
gnd	6-phosphogluconate dehydrogenase, decarboxylating
greA	Transcription elongation factor GreA
groL	60 kDa chaperonin
groS	10 kDa chaperonin
group_2128	Purine nucleoside phosphoramidase
group_2468	hypothetical protein
group_2517	hypothetical protein
group_2720	hypothetical protein

group_3140	hypothetical protein
group_3273	hypothetical protein
group_3367	N-carbamoyl-D-amino acid hydrolase
group_7397	hypothetical protein
grpE	Protein GrpE
grxA	Glutaredoxin 1
guaA	GMP synthase [glutamine-hydrolyzing]
guaB	Inosine-5'-monophosphate dehydrogenase
gyrB	DNA gyrase subunit B
hda	DnaA regulatory inactivator Hda
hemW	Heme chaperone HemW
hflC	Modulator of FtsH protease HflC
hisS	Histidine--tRNA ligase
hslU	ATP-dependent protease ATPase subunit HslU
hslV	ATP-dependent protease subunit HslV
htpG	Chaperone protein HtpG
ibaG	Acid stress protein IbaG
ileS	Isoleucine--tRNA ligase
infC	Translation initiation factor IF-3
iscS	Cysteine desulfurase IscS
ispG	4-hydroxy-3-methylbut-2-en-1-yl diphosphate synthase (flavodoxin)
ispH	4-hydroxy-3-methylbut-2-enyl diphosphate reductase
kdsA	2-dehydro-3-deoxyphosphooctonate aldolase
kdsB	3-deoxy-manno-octulosonate cytidylyltransferase
lepA	Elongation factor 4
lolE	Lipoprotein-releasing system transmembrane protein LolE
lon_1	Lon protease
lpdA	Dihydrolipoyl dehydrogenase
lptB	Lipopolysaccharide export system ATP-binding protein LptB
lptG	Lipopolysaccharide export system permease protein LptG
lpxA	Acyl-[acyl-carrier-protein]--UDP-N-acetylglucosamine O-acyltransferase
lpxC	UDP-3-O-acyl-N-acetylglucosamine deacetylase
lpxD	UDP-3-O-(3-hydroxymyristoyl)glucosamine N-acyltransferase
lpxL	Lipid A biosynthesis lauroyltransferase
lpxM	Lipid A biosynthesis myristoyltransferase
lysP	Lysine-specific permease
lysS	Lysine--tRNA ligase
map	Methionine aminopeptidase
mdfA_1	Multidrug transporter MdfA
mepM	Murein DD-endopeptidase MepM
metI	D-methionine transport system permease protein MetI
metK	S-adenosylmethionine synthase
mgtB	Magnesium-transporting ATPase, P-type 1
minD	Septum site-determining protein MinD
mnmG	tRNA uridine 5-carboxymethylaminomethyl modification enzyme MnmG
mraY	Phospho-N-acetylmuramoyl-pentapeptide-transferase
mreB	Cell shape-determining protein MreB

msbA	Lipid A export ATP-binding/permease protein MsbA
mscM	Miniconductance mechanosensitive channel MscM
mtnN	5'-methylthioadenosine/S-adenosylhomocysteine nucleosidase
murA	UDP-N-acetylglucosamine 1-carboxyvinyltransferase
murC	UDP-N-acetylmuramate--L-alanine ligase
murG	UDP-N-acetylglucosamine--N-acetylmuramyl-(pentapeptide) pyrophosphoryl-undecaprenol N-acetylglucosamine transferase
murJ_2	putative lipid II flippase MurJ
nadB	L-aspartate oxidase
nadE	Glutamine-dependent NAD(+) synthetase
nadK	NAD kinase
ndk	Nucleoside diphosphate kinase
nfuA	Fe/S biogenesis protein NfuA
nrdA	Ribonucleoside-diphosphate reductase 1 subunit alpha
nrdB	Ribonucleoside-diphosphate reductase 1 subunit beta
nrdR	Transcriptional repressor NrdR
nth	Endonuclease III
nuoB	NADH-quinone oxidoreductase subunit B
nuoC	NADH-quinone oxidoreductase subunit C/D
nuoF	NADH-quinone oxidoreductase subunit F
nuoI	NADH-quinone oxidoreductase subunit I
nuoK	NADH-quinone oxidoreductase subunit K
nusA	Transcription termination/antitermination protein NusA
nusB	Transcription antitermination protein NusB
nusG	Transcription termination/antitermination protein NusG
orn	Oligoribonuclease
parE	DNA topoisomerase 4 subunit B
pfkA	ATP-dependent 6-phosphofructokinase isozyme 1
pgi	Glucose-6-phosphate isomerase
phoU	Phosphate-specific transport system accessory protein PhoU
plsC	1-acyl-sn-glycerol-3-phosphate acyltransferase
pnp	Polyribonucleotide nucleotidyltransferase
ppa	Inorganic pyrophosphatase
prfA	Peptide chain release factor RF1
prfB	Peptide chain release factor RF2
proY	Proline-specific permease ProY
prs	Ribose-phosphate pyrophosphokinase
pstB	Phosphate import ATP-binding protein PstB
pstC	Phosphate transport system permease protein PstC
ptsI	Phosphoenolpyruvate-protein phosphotransferase
ptsN	Nitrogen regulatory protein
purA	Adenylosuccinate synthetase
purB	Adenylosuccinate lyase
purC	Phosphoribosylaminoimidazole-succinocarboxamide synthase
purE	N5-carboxyaminoimidazole ribonucleotide mutase
purF	Amidophosphoribosyltransferase
purM	Phosphoribosylformylglycinamidine cyclo-ligase
purU	Formyltetrahydrofolate deformylase

putP	Sodium/proline symporter
pykF	Pyruvate kinase I
pyrD	Dihydroorotate dehydrogenase (quinone)
pyrF	Orotidine 5'-phosphate decarboxylase
pyrG	CTP synthase
pyrH	Uridylate kinase
qmcA	Protein QmcA
radA	DNA repair protein RadA
recA	Protein RecA
rho	Transcription termination factor Rho
ribA	GTP cyclohydrolase-2
ribH	6,7-dimethyl-8-ribityllumazine synthase
rlmN	Dual-specificity RNA methyltransferase RlmN
rnb	Exoribonuclease 2
rnpA	Ribonuclease P protein component
rnt	Ribonuclease T
rpiA	Ribose-5-phosphate isomerase A
rplA	50S ribosomal protein L1
rplB	50S ribosomal protein L2
rplC	50S ribosomal protein L3
rplD	50S ribosomal protein L4
rplE	50S ribosomal protein L5
rplJ	50S ribosomal protein L10
rplL	50S ribosomal protein L7/L12
rplN	50S ribosomal protein L14
rplP	50S ribosomal protein L16
rplQ	50S ribosomal protein L17
rplS	50S ribosomal protein L19
rplT	50S ribosomal protein L20
rplU	50S ribosomal protein L21
rplV	50S ribosomal protein L22
rpmA	50S ribosomal protein L27
rpmB	50S ribosomal protein L28
rpmD	50S ribosomal protein L30
rpmF	50S ribosomal protein L32
rpmG	50S ribosomal protein L33
rpmH	50S ribosomal protein L34
rpmI	50S ribosomal protein L35
rpoA	DNA-directed RNA polymerase subunit alpha
rpoB	DNA-directed RNA polymerase subunit beta
rpoC	DNA-directed RNA polymerase subunit beta'
rpoD	RNA polymerase sigma factor RpoD
rpoE	ECF RNA polymerase sigma-E factor
rpoH	RNA polymerase sigma factor RpoH
rpsA	30S ribosomal protein S1
rpsB	30S ribosomal protein S2
rpsC	30S ribosomal protein S3

rpsD	30S ribosomal protein S4
rpsE	30S ribosomal protein S5
rpsG	30S ribosomal protein S7
rpsI	30S ribosomal protein S9
rpsJ	30S ribosomal protein S10
rpsK	30S ribosomal protein S11
rpsL	30S ribosomal protein S12
rpsO	30S ribosomal protein S15
rpsR	30S ribosomal protein S18
rpsT	30S ribosomal protein S20
rpsU	30S ribosomal protein S21
rseB	Sigma-E factor regulatory protein RseB
rsmB	Ribosomal RNA small subunit methyltransferase B
rsmG	Ribosomal RNA small subunit methyltransferase G
rsmH	Ribosomal RNA small subunit methyltransferase H
rsmI	Ribosomal RNA small subunit methyltransferase I
ruvA	Holliday junction ATP-dependent DNA helicase RuvA
ruvB	Holliday junction ATP-dependent DNA helicase RuvB
ruvC	Crossover junction endodeoxyribonuclease RuvC
sapD	Putrescine export system ATP-binding protein SapD
sdhA	Succinate dehydrogenase flavoprotein subunit
sdhC	Succinate dehydrogenase cytochrome b556 subunit
sdhD	Succinate dehydrogenase hydrophobic membrane anchor subunit
secA	Protein translocase subunit SecA
secE	Protein translocase subunit SecE
secF	Protein translocase subunit SecF
secY	Protein translocase subunit SecY
smpB	SsrA-binding protein
sohB	putative protease SohB
speD	S-adenosylmethionine decarboxylase proenzyme
spoT	Bifunctional (p)ppGpp synthase/hydrolase SpoT
sucB	Dihydrolipoyllysine-residue succinyltransferase component of 2-oxoglutarate dehydrogenase complex
sucC	Succinate--CoA ligase [ADP-forming] subunit beta
sucD	Succinate--CoA ligase [ADP-forming] subunit alpha
sufB	FeS cluster assembly protein SufB
sufC	putative ATP-dependent transporter SufC
sufE	Cysteine desulfuration protein SufE
suhB	Inositol-1-monophosphatase
talB	Transaldolase B
thiQ	Thiamine import ATP-binding protein ThiQ
thrB	Homoserine kinase
thrC	Threonine synthase
thyA	Thymidylate synthase
topA	DNA topoisomerase 1
tpiA	Triosephosphate isomerase
trkA	Trk system potassium uptake protein TrkA
trkH	Trk system potassium uptake protein TrkH

trmD	tRNA (guanine-N(1)-)-methyltransferase
trmJ	tRNA (cytidine/uridine-2'-O-)-methyltransferase TrmJ
trpS	Tryptophan--tRNA ligase
truB	tRNA pseudouridine synthase B
tsaD	tRNA N6-adenosine threonylcarbamoyltransferase
tusC	Protein TusC
tusD	Sulfurtransferase TusD
typA	GTP-binding protein TypA/BipA
tyrS	Tyrosine--tRNA ligase
ubiA	4-hydroxybenzoate octaprenyltransferase
ubiE	Ubiquinone/menaquinone biosynthesis C-methyltransferase UbiE
ubiH	2-octaprenyl-6-methoxyphenol hydroxylase
ubiX	Flavin prenyltransferase UbiX
usg	USG-1 protein
valS	Valine--tRNA ligase
xthA	Exodeoxyribonuclease III
yajC	Sec translocon accessory complex subunit YajC
ybaB	Nucleoid-associated protein YbaB
ybhL	Inner membrane protein YbhL
ydjM	Inner membrane protein YdjM
yecS	hypothetical protein
yfiA	Ribosome-associated factor Y
ymoA	Modulating protein YmoA
znuB	High-affinity zinc uptake system membrane protein ZnuB
znuC	Zinc import ATP-binding protein ZnuC

Table S3: Accession numbers for genomes used to produce phylogeny

***H. defensa* isolates used to infer phylogeny**	**WGS project no.**	**Genbank assembly no.**
HaCiconfinis_2801_v1.0	CADIJI01	GCA_902859675.1
HaCicuneomaculata-2628	CADIJH01	GCA_902859575.1
B. tabaci-MED	AJLH02	GCA_000258345.1
B. tabaci-MEAM1	CP016303.1	GCA_002285855.1
A. pisum-5DH	CP021663-CP021668	GCA_003122445.1
A.pisum-A2C	CP017606- CP017609	GCA_002777195.1
A.pisum-ZA17	CP017613-CP017616	GCA_002777235.1
A.pisum-AS3	CP017610-CP017612	GCA_002777215.1
A.pisum-NY26	CP017605.1	GCA_002777295.1
A.pisum-MI47	CP022932-CP022937	GCA_002269405.1
A.pisum-MI12	CP023987-CP023990	GCA_003590545.1
A.pisum-SC_495	JAABOV01	GCA_016635095.1
**Including genomes produced in this study**		
A.pisum-3492	CP133792-CP133796	
A.pisum-2185	CP133493-CP133498	
A.pisum-I928	CP133801-CP133803	

Table S4: Generalized linear model results for parasitism assays.

A. *Hamiltonella defensa* C11 isolates, standard assay at 20°C

**Aphid survival** (AA vs M + DM)		**Whole model per parasitoid**<br>Pp GzLM: N= 450, χ^2 = 21.6 df = 2, P <0.0001<br>Ae GzLM: N = 373, χ^2 = 0.003 df = 2, P = 0.96	**Pearson overdispersion**<br>χ^2/df = 1.0007; deviance/df = 1.0<br>χ^2/df = 1.0005; deviance/df = .955
**Contrast**	**Challenge**		**Odds Ratios (95% CI)**
2185→5D (no APSE) vs 5D control	*Praon pequodorum*	χ^2 = 0.002 P = 0.96	1.0 (0.69 – 1.44)
3492→5D vs 5D control	*Praon pequodorum*	**χ^2 = 16.2 P <0.0001**	1.80 (1.33 -2.28)<br>80% increase in odds of aphid survival
3492→5D vs 5D control	*Aphidius ervi*	χ^2 = 0.003 P = 0.96	1.01 (0.77 – 1.31)

**Wasp survival** (M vs AA + DM)		**Whole model per parasitoid**<br>Pp GzLM: N= 450, χ^2 = 26.7 df = 2, P <0.0001<br>Ae GzLM: N = 373, χ^2 = 0.18, df = 1, P = 0.67	**Pearson overdispersion**<br>χ^2/df = 1.007; deviance/df = 1.31<br>χ^2/df = 1.005; deviance/df = 1.20
**Contrast**	**Challenge**		**Odds Ratios (95%)**
2185→5D (no APSE) vs 5D control	*Praon pequodorum*	χ^2 = 1.29 P = 0.26	1.16 (0.90 – 1.48)
3492→5D vs 5D control	*Praon pequodorum*	**χ^2 = 15.4 P < 0.0001**	0.64 (0.51 -0.81)<br>36% decrease in odds of wasp survival
3492→5D vs 5D control	*Aphidius ervi*	χ^2 = 0.18 P = 0.67	0.96 (0.76 – 1.19)

**Dual mortality (aphid & wasp perish)**<br>(DM vs AA + M)		**Whole model per parasitoid**<br>GzLM: N= 450, χ^2 = 4.15 df = 2, P =0.13<br>Ae GzLM: N = 373, χ^2 = 0.32 df = 1, P = 0.57	**Pearson overdispersion**<br>χ^2/df = 1.007, deviance/df = 1.0<br>χ^2/df = 1.005; deviance/df = 1.0
**Contrast**	**Challenge**		**Odds Ratios (95% CI)**
2185→5D vs 5D control	*Praon pequodorum*	χ^2 = 1.6 P = 0.21	0.83 (0.62 to 1.11)
3492→5D vs 5D control	*Praon pequodorum*	χ^2 = 0.7 P = 0.41	1.11 (0.86 to 1.43)
3492→5D vs 5D control	*Aphidius ervi*	χ^2 = 0.18 P = 0.67	1.10 (0.79 – 1.52)

B. *Hamiltonella defensa* C9 isolates, standard assay at 20°C

	Whole model per parasitoid		Pearson overdispersion
**Aphid survival** (AA vs M + DM)	Pp GzLM: n = 303, χ^2 = 2.34 df = 2, *P* = 0.31<br>Ae GzLM: n = 622, χ^2 = 215.9 df = 3, *P* = 0.31		χ^2/df = 1.010, deviance/df = 0.73<br>χ^2/df = 1.007, deviance/df = 1.03
**Contrast**	**Challenge**		**Odds Ratios (95% CI)**
I928→ND18 vs ND18 control	*Praon pequodorum*	χ^2 = 0.005 *P* = 0.94	1.0 (0.64–1.57)
3483→ND18 vs ND18 control	*Praon pequodorum*	χ^2 = 1.81 *P* = 0.18	1.29 (0.88–1.89)
I928→ND18 vs ND18 control	*Aphidius ervi*	**χ^2 = 176.5 *P* < 0.0001**	5.86 (4.28–8.02)<br>486% increase in odds of aphid survival
3483→ND18 vs ND18 control	*Aphidius ervi*	**χ^2 = 144.8 *P* < 0.0001**	5.13 (3.82–6.90)<br>413 % increase in odds of aphid survival
4721→ND18 vs ND18 control	*Aphidius ervi*	**χ^2 = 71.1 *P* < 0.0001**	3.01 (2.28–3.97)<br>201% increase in odds of aphid survival

		Whole model per parasitoid	Pearson overdispersion
**Wasp survival** (M vs AA + DM)		Pp GzLM: n= 303, χ^2 = 4.1, df =2, *P* = 0.12<br>Ae GzLM: n= 622, χ^2 = 327.1, df = 3, *P* < 0.0001 *Firth bias adjusted	χ^2/df = 1.010, 1.005; deviance/df = 1.23<br>χ^2/df = 0.75, 1.005; deviance/df = 0.5
**Contrast**	**Challenge**		**Odds Ratios (95% CI)**
I928→ND18 vs ND18 Control	*Praon pequodorum*	χ^2 = 3.38 *P* = 0.07	0.75 (0.56–1.02)
3483→ND18 vs ND18 control	*Praon pequodorum*	χ^2 = 2.86 *P* = 0.09	0.77 (0.7–1.04)
I928→ND18 vs ND18 control	*Aphidius ervi*	**χ^2 = 190.4 *P* < 0.0001**	0.08 (0.06–0.12)<br>92% decrease in odds of wasp survival
3483→ND18 vs ND18 control	*Aphidius ervi*	**χ^2 = 208.3 *P* < 0.0001**	0.10 (0.07–0.15)<br>90% decrease in odds of wasp survival
4721→ND18 vs ND18 control	*Aphidius ervi*	**χ^2 = 163.2 *P* < 0.0001**	0.17 (0.12–0.23)<br>83% decrease in odds of wasp survival

Table S4B Continued

**Dual mortality (aphid & wasp perish)** (DM vs AA + M)		**Whole model per parasitoid** GzLM: n= 303, χ^2 = 31.8 df = 2, P < 0.001<br>GzLM: n= 622, χ^2 = 31.1 df = 3, P < 0.001	**Pearson overdispersion** χ^2/df = 0.999, deviance/df = 0.97<br>χ^2/df = 1.007, deviance/df = 1.04
**Contrast**	**Challenge**		**Odds Ratios (95% CI)**
I928→ND18 vs ND18 Control	*Praon pequodorum*	χ^2 = 4.29 P = 0.04	1.44 (1.01 to 2.07)<br>Marginally significant increase in odds of dual mortality
3483→ND18 vs ND18 control	*Praon pequodorum*	χ^2 = 0.73 P = 0.39	1.18 (0.95 to 1.88)
I928→ND18 vs ND18 Control	*Aphidius ervi*	χ^2 = 20.13 P = 0.72	0.95 (0.71 – 1.26)
3483→5D-ND18 vs ND18 control	*Aphidius ervi*	χ^2 = 1.7 P = 0.19	1.21 (0.93-1.58)
4721→ND18 vs ND18 control	*Aphidius ervi*	**χ^2 = 1.81 P < 0.0001**	1.81 (1.4 – 2.34)<br>88% increase in odds of dual mortality

C. *Hamiltonella defensa* C11 isolates, 'cool' vs 'heat wave' assay

**Aphid survival** (AA vs M + DM)		**Whole model per parasitoid** Pp GzLM: n = 377, χ^2 = 84.8 df = 3, P < 0.0001<br>Ae GzLM: n = 586, χ^2 = 2.2 df = 3, P = 053	**Pearson overdispersion** χ^2/df = 1.011; deviance/df = 1.03<br>χ^2/df = 1.007; deviance/df = 0.63
**Contrast**	**Challenge**		**Odds Ratios (95% CI)**
3492→5D cool vs 5D cool	*Praon pequodorum*	**χ^2 = 35.23 *P* < 0.0001**	2.91 (1.9–4.30)<br>191% increase in odds of aphid survival
3492→5D heat wave vs 5D cool	*Praon pequodorum*	**χ^2 = 25.17 *P* < 0.0001**	2.35 (1.58–3.50)<br>135% increase in odds of aphid survival
5D heat wave vs 5D cool	*Praon pequodorum*	χ^2 = 2.30 P = 0.13	0.68 (0.48–0.97)
3492→5D cool vs 5D cool	*Aphidius ervi*	χ^2 = 0.35 P = 0.56	0.84 (0.57–1.24)
3492→5D heat wave vs 5D cool	*Aphidius ervi*	χ^2 = 0.07 P = 0.78	1.12 (0.74–1.69)
5D heat wave vs 5D cool	*Aphidius ervi*	χ^2 = 0.69 P = 0.41	1.05 (0.70–1.59)

Table S4C continued

**Wasp survival**<br>(M vs AA + DM)		**Whole model per parasitoid**<br>Pp GzLM: n = 381, χ^2 = 103.8 df = 3, P < 0.0001<br>Ae GzLM: n = 586, χ^2 = 39.6 df = 3, P < 0.0001	**Pearson overdispersion**<br>χ^2/df = 1.011; deviance/df = 0.999<br>χ^2/df = 1.007; deviance/df = 1.33
**Contrast**	**Challenge**		**Odds Ratios (95% CI)**
3492→5D cool vs 5D cool	*Praon pequodorum*	**χ^2 = 43.5 P < 0.0001**	0.29 (0.18–0.46)<br>71% decrease in odds of wasp survival
3492→5D heat wave vs 5D cool	*Praon pequodorum*	**χ^2 = 53.7 P < 0.0001**	0.29 (0.19–0.45)<br>71.0% decrease in odds of wasp survival
5D heat wave vs 5D cool	*Praon pequodorum*	χ^2 = 0.001 P = 0.99	1.0 (0.59-1.69)
3492→5D cool vs 5D cool	*Aphidius ervi*	χ^2 = 0.07 P = 0.79	0.04 (0.01–0.15)
3492→5D heat wave vs 5D cool	*Aphidius ervi*	χ^2 = 0.58 P = 0.44	0.17 (0.12–0.24)
5D heat wave vs 5D cool	*Aphidius ervi*	**χ^2 = 23.9 P < 0.0001**	0.56 (0.44–0.73)<br>44% decrease in odds of wasp survival

**Dual mortality (aphid & wasp perish)**<br>(DM vs AA + M)		**Whole model per parasitoid**<br>Pp GzLM: n = 381, χ^2 = 5.1 df =3, P = 0.17<br>Ae GxLM: n = 586 χ^2 = 50.7 df =3, P < 0.0001	**Pearson overdispersion**<br>χ^2/df = 1.011; deviance/df = 1.31<br>χ^2/df = 1.007; deviance/df = 1.26
**Contrast**	**Challenge**		**Odds Ratios (95% CI)**
3492→5D cool vs 5D cool	*Praon pequodorum*	χ^2 = 2.38 P = 0.12	1.09 (0.78–1.53)
3492→5D heat wave vs 5D cool	*Praon pequodorum*	χ^2 = 4.42 P = 0.04	1.48 (1.03–2.13)<br>Marginally significant increase in odds of dual mortality
5D heat wave vs 5D cool	*Praon pequodorum*	χ^2 = 0.97 P = 0.32	1.17 (0.84–1.62)
3492→5D cool vs 5D cool	*Aphidius ervi*	χ^2 = 0.01 P = 0.92	0.99 (0.73–1.34)
3492→5D heat wave vs 5D cool	*Aphidius ervi*	χ^2 = 1.0 P = 0.32	0.89 (0.68 –1.16)
5D heat wave vs 5D cool	*Aphidius ervi*	**χ^2 = 28.3 P < 0.0001**	1.87 (1.39 –2.52)<br>87% increase in odds of dual mortality

D. *Hamiltonella defensa* C9 isolates, 'cool' vs 'heat wave' assay

**Aphid survival** (AA vs M + DM)		**Whole model**<br>Ae GzLM: n = 1261 χ^2 = 774.9 df = 5, P < 0.0001	**Pearson overdispersion**<br>χ^2/df = 1.005; deviance/df = 0.98

Contrast	Challenge		Odds Ratios (95% CI)
3483→ND18 cool vs ND18 cool	*Aphidius ervi*	**χ^2 = 295.5 *P* < 0.0001**	5.05 (4.10–6.32)<br>367% increase in aphid survival
3483→ND18 heat wave vs ND18 cool	*Aphidius ervi*	**χ^2 = 63.3 *P* < 0 .0001**	2.44 (1.95–3.05)<br>144% increase in aphid survival
I928→ND18 cool vs ND18 cool	*Aphidius ervi*	**χ^2 = 183.2 *P* < 0.0001**	4.46 (3.62–5.50)<br>346% increase in aphid survival
I928→ND18 heat wave vs ND18 cool	*Aphidius ervi*	**χ^2 = 239.5 *P* < 0.0001**	5.76 (4.58–7.24)<br>476% increase in aphid survival
5D heat wave vs ND18 cool	*Aphidius ervi*	χ^2 = 0.06 *P* = 0.81	1.01 (0.73–1.39)

**Wasp survival**<br>(M vs AA + DM)		**Whole model**<br>Ae GzLM: n = 1261, χ^2 = 39.6 df = 3, P < 0.0001 *Firth bias adjusted	**Pearson overdispersion**<br>χ^2/df = 0.737 P= 0.99

Contrast	Challenge		Odds Ratios (95% CI)
3483→ND18 cool vs ND18 cool	*Aphidius ervi*	**χ^2 =553.7 *P* < 0.0001**	0.03 (0.002–0.23)<br>97.5% decrease in odds of wasp survival
3483→ND18 heat wave vs ND18 cool	*Aphidius ervi*	**χ^2 =215.6 *P* < 0.0001**	0.16 (0.03–0.61)<br>84 % decrease in odds of wasp survival
I928→ND18 cool vs ND18 cool	*Aphidius ervi*	**χ^2 = 224.4 *P* < 0.0001**	0.13 (0.03–0.48)<br>87% decrease in odds of wasp survival
I928→ND18 heat wave vs ND18 cool	*Aphidius ervi*	**χ^2 = 263.0 *P* < 0.0001**	0.09 (0.02–0.38)<br>91% decrease in odds of wasp survival
5D heat wave vs ND18 cool	*Aphidius ervi*	χ^2 = 0.11 *P* = 0.57	0.94 (0.7–1.24)

Table S4D continued

**Dual mortality (aphid & wasp perish)** (DM vs AA + M)		**Whole model** Ae GxLM: n = 1261 $\chi^2 = 69.5$ df =5, $P < 0.0001$	**Pearson overdispersion** $\chi^2/df = 1.005$;
**Contrast**	**Challenge**		**Odds Ratios (95% CI)**
3483→ND18 cool vs ND18 cool	*Aphidius ervi*	$\chi^2 = 4.4$ $P = 0.04$	0.19 (0.04–0.94) Marginally significant decrease in odds of dual mortality
3483→ND18 heat wave vs ND18 cool	*Aphidius ervi*	$\boldsymbol{\chi^2 = 57.8}$ $\boldsymbol{P < 0.0001}$	2.28 (1.43–3.63) 128% increase in odds of dual mortality
I928→ND18 cool vs ND18 cool	*Aphidius ervi*	$\chi^2 = 2.67$ $P = 0.10$	1.23 (0.04–35.6)
I928→ND18 heat wave vs ND18 cool	*Aphidius ervi*	$\chi^2 = 0.03$ $P = 0.84$	1.03 (0.28–3.77)
5D heat wave vs ND18 cool	*Aphidius ervi*	$\chi^2 = 0.13$ $P = 0.64$	1.06 (0.2 -5.66)

Table S5: CDS differences among C-clade *H. defensa* strains

A. CDS occurring in I928 and MI12 C9 *H. defensa,* but not present in C11 *H. defensa*

location	C9-MI12 locus tag	C9-I928 locus tag	CDS name	most similar blast hit	coverage	% similarity
pHDMI12.p1/pHDI928.1	CJJ19_10750		hypothetical protein	other Hamiltonella defensa	98	99
pHDMI12.p1/pHDI928.1	CJJ19_10805	REQ42_10560	hypothetical protein	other Hamiltonella defensa	98	97
pHDMI12.p1/pHDI928.1	CJJ19_10855	REQ42_10591	replication regulatory protein RepA	other Hamiltonella defensa	69	99
pHDMI12.p1/pHDI928.1	CJJ19_10865	REQ42_10605	hypothetical protein	other Hamiltonella defensa	100	92
pHDMI12.p1/pHDI928.1	CJJ19_10875	REQ42_10615	ParB/RepB/Spo0J family partition protein	Photorhabdus sp. RM71S	94	99
pHDMI12.p1/pHDI928.1	CJJ19_10880	REQ42_10620	helix-turn-helix domain-containing protein	other Hamiltonella defensa	99	100
pHDMI12.p1/pHDI928.1	CJJ19_10885	REQ42_10625	FlhC family transcriptional regulator	Salmonella enterica	96	97
pHDMI12.p1/pHDI928.1	CJJ19_10890	REQ42_10630	transcriptional regulator	other Hamiltonella defensa	100	100
pHDMI12.p1/pHDI928.1	CJJ19_10895	REQ42_10635	transglycosylase	other Hamiltonella defensa	100	100
pHDMI12.p1/pHDI928.1	CJJ19_10900	REQ42_10640	hypothetical protein	other Hamiltonella defensa	100	100
pHDMI12.p1/pHDI928.1	CJJ19_10910	REQ42_10650	conjugal transfer protein TraG	other Hamiltonella defensa	99	100

pHDMI12.p1/pHDI928.1	CJJ19_10915	REQ42_10655	conjugal transfer protein TraH	other Hamiltonella defensa	100	100
pHDMI12.p1/pHDI928.1	CJJ19_10920	REQ42_10660	conjugal transfer protein TraF	other Hamiltonella defensa	100	100
pHDMI12.p1/pHDI928.1	CJJ19_10925	REQ42_10665	protein-disulfide isomerase	other Hamiltonella defensa	100	100
pHDMI12.p1/pHDI928.1	CJJ19_10930	REQ42_10670	transposase	other Hamiltonella defensa	100	100
pHDMI12.p1/pHDI928.1	CJJ19_10935	REQ42_10685	hypothetical protein	other Hamiltonella defensa	100	100
pHDMI12.p1/pHDI928.1	CJJ19_10940	REQ42_10690	hypothetical protein	other Hamiltonella defensa	100	100
pHDMI12.p1/pHDI928.1	CJJ19_10945	REQ42_10695	DNA-binding protein	other Hamiltonella defensa	100	100
pHDMI12.p1/pHDI928.1	CJJ19_10955	REQ42_10705	conjugal transfer protein	other Hamiltonella defensa	100	100
pHDMI12.p1/pHDI928.1	CJJ19_10960	REQ42_10710	conjugal transfer protein	other Hamiltonella defensa	100	100
pHDMI12.p1/pHDI928.1	CJJ19_10965	REQ42_10715	hypothetical protein	other Hamiltonella defensa	100	100
pHDMI12.p1/pHDI928.1	CJJ19_10970	REQ42_10720	conjugal transfer protein	other Hamiltonella defensa	100	100
pHDMI12.p1/pHDI928.1	CJJ19_10975	REQ42_10721	hypothetical protein	other Hamiltonella defensa	100	100
pHDMI12.p1/pHDI928.1	CJJ19_10980	REQ42_10725	traL	other Hamiltonella defensa	100	100
pHDMI12.p1/pHDI928.1	CJJ19_10985	REQ42_10730	pilus assembly protein	other Hamiltonella defensa	100	100
pHDMI12.p1/pHDI928.1	CJJ19_10990	REQ42_10735	conjugal transfer protein TraK	other Hamiltonella defensa	100	100
pHDMI12.p1/pHDI928.1	CJJ19_10995	REQ42_10740	conjugal transfer protein TraB CDS	other Hamiltonella defensa	100	100
pHDMI12.p1/pHDI928.1	CJJ19_11000	REQ42_10745	traV CDS	other Hamiltonella defensa	100	100
pHDMI12.p1/pHDI928.1	CJJ19_11005	REQ42_10750	conjugal transfer protein TraA CDS	other Hamiltonella defensa	100	100
pHDMI12.p1/pHDI928.1	CJJ19_11015	REQ42_10760	traC CDS	other Hamiltonella defensa	100	100
pHDMI12.p1/pHDI928.1	CJJ19_11020	REQ42_10765	hypothetical protein CDS	other Hamiltonella defensa	100	100
pHDMI12.p1/pHDI928.1	CJJ19_11025	REQ42_10770	S26 family signal peptidase CDS	other Hamiltonella defensa	100	100
pHDMI12.p1/pHDI928.1	CJJ19_11030	REQ42_10775	conjugal transfer protein TraW CDS	other Hamiltonella defensa	100	100
pHDMI12.p1/pHDI928.1	CJJ19_11035	REQ42_10780	diguanylate phosphodiesterase CDS	other Hamiltonella defensa	100	100
pHDMI12.p1/pHDI928.1	CJJ19_11040	REQ42_10785	conjugal transfer protein TraU CDS	other Hamiltonella defensa	100	100
pHDMI12.p1/pHDI928.1	CJJ19_11045	REQ42_10790	conjugal transfer protein TraN CDS	other Hamiltonella defensa	100	100
pHDMI12.p1/pHDI928.1	CJJ19_11055	REQ42_10795	hypothetical protein CDS	other Hamiltonella defensa	100	100
pHDMI12.p1/pHDI928.1	CJJ19_11060	REQ42_10800	hypothetical protein CDS	other Hamiltonella defensa	100	100
pHDMI12.p1/pHDI928.1	CJJ19_11065	REQ42_10805	hypothetical protein CDS	other Hamiltonella defensa	100	100
pHDMI12.p1/pHDI928.1	CJJ19_11070	REQ42_10806	hypothetical protein CDS	other Hamiltonella defensa	100	100
pHDMI12.p1/pHDI928.1	CJJ19_11075	REQ42_10815	hypothetical protein CDS	other Hamiltonella defensa	100	100
pHDMI12.p1/pHDI928.1	CJJ19_11135	REQ42_10815	hypothetical protein CDS	other Hamiltonella defensa	100	100
pHDMI12.p1/pHDI928.1	CJJ19_11140	REQ42_10811	hypothetical protein CDS	other Hamiltonella defensa	100	100
pHDMI12.p1/pHDI928.1	CJJ19_11145	REQ42_10805	hypothetical protein CDS	other Hamiltonella defensa	100	100
pHDMI12.p1/pHDI928.1	CJJ19_11150	REQ42_10805	hypothetical protein CDS	other Hamiltonella defensa	100	100
chromosome	CJJ19_07260	REQ42_07290	hypothetical protein	other Hamiltonella defensa	92	82
chromosome	CJJ19_03785	REQ42_01505	hypothetical protein CDS	other Hamiltonella defensa		
chromosome	CJJ19_03790	REQ42_01500	DNA-binding protein CDS	other Hamiltonella defensa		
chromosome	CJJ19_03825	REQ42_01465	hypothetical protein CDS	other Hamiltonella defensa		

B. CDS occurring in 3492 C11 *H. defensa,* but not present in I928 and MI12 C9 *H. defensa*.

location	C11-3492 locus tag	CDS name	most similar blast hit	Coverage (%)	similarity (%)
pHD3492.2	REO43_11305	replication/maintenance protein RepL CDS	other Hamiltonella defensa		
pHD3492.2	REO43_11335	hypothetical protein CDS	other Hamiltonella defensa		
pHD3492.2	REO43_11365	hypothetical protein CDS	other Hamiltonella defensa		
pHD3492.2	REO43_10715	transposase CDS	Regiella insecticola		
pHD3492.2	REO43_10730	type II toxin-antitoxin system RelE/ParE	Escherichia coli	99	87%
pHD3492.2	REO43_10735	XRE family transcriptional regulator CDS	other Hamiltonella defensa	99	100
pHD3492.2	REO43_10740	traM CDS	other Hamiltonella defensa	99	100
pHD3492.2	REO43_10760	IS630 family transposase CDS	other Hamiltonella defensa	98	72
pHD3492.2	REO43_10775	hypothetical protein CDS	other Hamiltonella defensa	84	100
pHD3492.2	REO43_10780	transposase CDS	other Hamiltonella defensa	98	100
pHD3492.2	REO43_10790	hypothetical protein CDS	other Hamiltonella defensa	100	98
pHD3492.2	REO43_10795	autotransporter outer membrane	other Hamiltonella defensa		99
pHD3492.2	REO43_10800	IS481 family transposase CDS	Acidovorax sp.	87	50
pHD3492.2	REO43_10805	hypothetical protein CDS	other Hamiltonella defensa	99	99
pHD3492.2	REO43_10880	hypothetical protein CDS	Arsenophonus nasoniae	97	81
pHD3492.2	REO43_10930	hypothetical protein CDS	Regiella insecticola	97	50
pHD3492.2	REO43_10935	MFS transporter CDS	Yersinia enterocolitica	99	55
pHD3492.2	REO43_10940	VOC family protein CDS	Vibrio profundum	96	50
pHD3492.2	REO43_10945	hypothetical protein CDS	Yersinia enterocolitica	99	49
pHD3492.2	REO43_10950	TraY domain-containing protein CDS	other Hamiltonella defensa	94	82
pHD3492.2	REO43_10955	hypothetical protein CDS	other Hamiltonella defensa	99	100
chromosome	REO43_03555	hypothetical protein	Veronia nyctiphanis		
chromosome	REO43_03560	hypothetical protein	Candidatus Vondammii		
chromosome	REO43_08995	AlpA family phage regulatory protein CDS	Candidatus Symbiopectobacterium	98	94
chromosome	REO43_09000	hypothetical protein CDS	other Hamiltonella defensa	97	43
chromosome	REO43_09095	phage major capsid protein CDS	other Hamiltonella defensa	100	99
chromosome	REO43_09100	HK97 family phage prohead protease	other Hamiltonella defensa	100	100
chromosome	REO43_09120	type II toxin-antitoxin system RelE/ParE family toxin	other Hamiltonella defensa	99	100
chromosome	REO43_09125	HigA family addiction module antitoxin CDS	other Hamiltonella defensa	99	100
chromosome	REO43_09140	hypothetical protein	Arsenophonus sp.	98	79
chromosome	REO43_09145	DUF551 domain-containing protein CDS	Arsenophonus sp.	99	75
chromosome	REO43_09200	hypothetical protein CDS	Arsenophonus sp.	84	49
chromosome	REO43_09240	hypothetical protein CDS	Plautia stali symbiont	99	92
chromosome	REO43_09245	DUF4145 domain-containing protein CDS	Arsenophonus sp.	100	76
chromosome	REO43_09270	hypothetical protein CDS	other Hamiltonella defensa		
